# Capture‐SELEX for a short aptamer for label‐free detection of salicylic acid

**DOI:** 10.1002/smo.20230007

**Published:** 2023-08-28

**Authors:** Lide Gu, Hanxiao Zhang, Yuzhe Ding, Yao Zhang, Deli Wang, Juewen Liu

**Affiliations:** ^1^ State Key Laboratory of Marine Environmental Science Xiamen University Xiamen Fujian China; ^2^ College of Ocean and Earth Sciences Xiamen University Xiamen Fujian China; ^3^ Department of Chemistry Waterloo Institute for Nanotechnology University of Waterloo Waterloo Ontario Canada

**Keywords:** aptamers, biosensor, capture‐SELEX, food detection, salicylic acid

## Abstract

Salicylic acid (SA) is a hydrolysis product and an active form of aspirin, and SA is found in a range of fruits and other food products. For food and drug and analysis there is a strong desire to detect SA. Since SA is a very small molecule, aptamers have advantages over antibodies for its detection. In this work, we used the library‐immobilization capture‐SELEX method to isolate aptamers for SA. After 17 rounds of selection, two main families of aptamers were isolated. The SA1 aptamer from family 1 has a *K*_*d*_ of 5.8 μM from a thioflavin T (ThT) fluorescence assay and 26.7 μM from isothermal titration calorimetry. The binding of other sequences was weaker compared to SA1. Based on mutation studies, the two conserved regions of SA1 were connected by two stems. Using ThT as a stain, a label‐free fluorescent sensor was tested for the detection of SA with a detection limit of 2.2 μM. A few similar molecules were tested including aspirin, and only p‐hydroxybenzoic acid showed a weak binding, indicating the high specificity of the SA1 aptamer. Finally, the SA1 aptamer was also tested in tomato juice and a similar binding performance was achieved.

## Graphical abstract

A short 40‐nucleotide aptamer was isolated using the library‐immobilization SELEX method, and this aptamer can selectively bind to salicylic acid but showed no binding to aspirin. A label‐free fluorescent sensor was developed using thioflavin T as a dye probe.

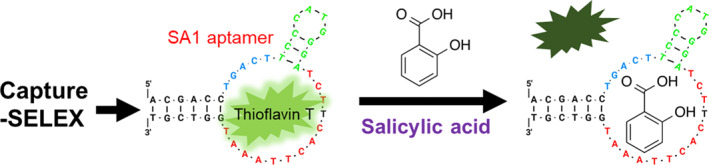

## 1 INTRODUCTION

Salicylic acid (SA) is the hydrolysis product of acetylsalicylic acid or aspirin, which is one of the most commonly used drugs for treating a diverse range of diseases.^[1]^ Because of its importance in drug and food chemistry, many methods have been developed to measure SA. Most of the detection methods relied on high performance liquid chromatography, which requires a lengthy sample treatment, operation and data analysis.[^2^, ^3^, ^4^] Although simple colorimetric tests were developed using metal salts such as ferric chloride,^[5]^ such methods lack sensitivity or specificity. Some recent work also used electrochemistry,^[6]^ and surface‐enhanced Raman spectroscopy.^[7]^ These methods rely on the intrinsic redox and molecular vibrational properties of SA, and are susceptible to interference in complex sample matrix. In terms of affinity ligands, SA binding proteins and antibodies are available,^[8]^ but few researchers have used them for SA detection.^[9]^ Immunoassays for small molecules often require competitive assays, which are less sensitive and require labeled target molecules.

DNA aptamers are single‐stranded DNA oligonucleotides isolated from in vitro selection and can bind to their targets with high affinity and specificity.[^10^, ^11^, ^12^, ^13^] Compared to antibodies, aptamers are more cost‐effective and stable. Aptamer for SA was previously reported using the library immobilization method.^[14]^ This aptamer was claimed to have two binding sites with *K*_*d*_ values of 34.6 nM and 4.7 μM, respectively. However, this aptamer was a 91‐mer sequence and truncation was not performed. Based on the predicted secondary structure, it is difficult to perform rational truncation, making it difficult to perform rational biosensor design.[^15^, ^16^, ^17^]

Using a hybridization‐based library‐immobilization method (also known as capture‐SELEX),[^18^, ^19^] many short aptamers were obtained.[^20^, ^21^, ^22^] In this work, we used the capture‐SELEX method to select new aptamers for SA and obtained a much shorter sequence with less than half the length of the previously reported aptamer. This sequence can be used as a smart molecule to respond to SA.

## 2 MATERIALS AND METHODS

### 2.1 Chemicals

All DNA oligonucleotides were synthesized by Integrated DNA Technologies (Coralville, IA) and sequences used in this work are listed in Supporting Information (SI) Table S1. Streptavidin‐coated agarose resin was purchased from Thermo Scientific (IL, USA). Salicylic acid, aspirin, benzoic acid, phenol, p‐hydroxybenzoic acid, catechol, phthalic acid, tyrosine, sodium chloride, magnesium chloride, sodium hydroxide, and hydrochloric acid were from Sigma‐Aldrich (MO, USA). 2‐(4‐Morpholino)ethanesulfonic acid (MES) was obtained from Biobasic Inc (Markham, ON, Canada). Ultra‐0.5 centrifugal filter units (3 *k* and 10 *k* molecular weight cut‐off) were purchased from Millipore‐Sigma (Oakville, ON, Canada). Micro bio‐spin chromatography columns and SsoFast EvaGreen supermix were from Bio‐Rad. dNTP mix, Taq DNA polymerase with ThermoPol buffer, and low molecular‐weight DNA ladder were purchased from New England Biolabs (Ipswich, MA).

### 2.2 SELEX

The SELEX experiment was carried out following previously described procedures,[^23^, ^24^] and detailed methods will not be repeated here. The selection buffer contained 10 mM MES, 100 mM NaCl, 5 mM MgCl_2_, pH 6. A SA stock solution was prepared in the selection buffer at a concentration of 10 mM and it was used for the entire selection experiment. The concentration of SA was 1 mM from round 1 to round 14, and 0.2 mM from round 15 to round 17. The polymerase chain reaction (PCR) products of round 14 and round 17 were sent for sequencing in the facility at the McMaster University.

### 2.3 ThT‐based assays

The measurement of thioflavin T (ThT) fluorescence spectra was carried out using a Variant Eclipse fluorescence spectrophotometer. An aptamer concentration of 1 μM and a ThT concentration of 2 μM were dissolved in 500 μL buffer (10 mM MES, pH 6, 100 mM NaCl, 5 mM MgCl_2_). The solution was transferred to a quartz cuvette and SA was gradually titrated to a final concentration of 30 μM. The excitation wavelength was set at 440 nm, and the emission was monitored from 460 to 510 nm. The fluorescence values at 490 nm were used for calculations. The *K*_*d*_ value was fitted using the following equation: *F* = *F*_0_ + *aK*_*d*_/(*K*_*d*_ + *x*), where *x* is the concentration of the titrated target, and *a* is the maximal signal change upon saturated binding.

### 2.4 Isothermal titration calorimetry

Isothermal titration calorimetry (ITC) was carried out using a MicroCal VP‐ITC. Aptamer and SA were respectively dissolved in SELEX buffer and degassed for 5 min prior to loading. Then, 281 μL SA solution (1 mM) was filled into the syringe and 1.45 mL aptamer (20 μM) was injected into the cell chamber. Except for an initial injection of 0.5 μL, 10 μL of the target was titrated into the cell each time over 20 s duration for a total of 28 injections at 25°C. Titration spacing was set for 360 s between each injection. The binding constant was obtained by fitting the titration curve to a one‐site binding model using the Origin software.^[25]^

### 2.5 Real sample test

Fresh tomatoes were purchased from a local supermarket and mashed by squeezing. The solid matter was removed by centrifugation. Then, the supernatant was collected and stored in a −20°C freezer for use. For real sample test, 10% raw tomato juice was added in the selection buffer and mixed with 1 μM SA1 aptamer and 2 μM ThT. Finally, the fluorescence intensity was measured as the initial signal, and then a series of standard SA solutions were injected into the above mixture to achieve designated final concentrations. The fluorescence spectra were recorded for analysis.

## 3 RESULTS AND DISCUSSION

### 3.1 Selection of Salicylic acid binding aptamers and sequence analysis

The aptamer selection followed our previously published method by immobilization of a DNA library containing a 30‐nucleotide random region (N30) on a streptavidin column via hybridization to a biotinylated DNA.[^20^, ^23^] Flanking the N30 region are two fixed primer binding regions for PCR amplification, and these regions can form base pairs to facilitate the release of aptamer sequences from the column upon binding to SA. In the selection process, the SA concentration was fixed at 1 mM for the first 14 rounds and reduced to 0.2 mM in the last 3 rounds. The selection was stopped at round 17, and the round 14 and 17 PCR products were sequenced.

**FIGURE 1:**
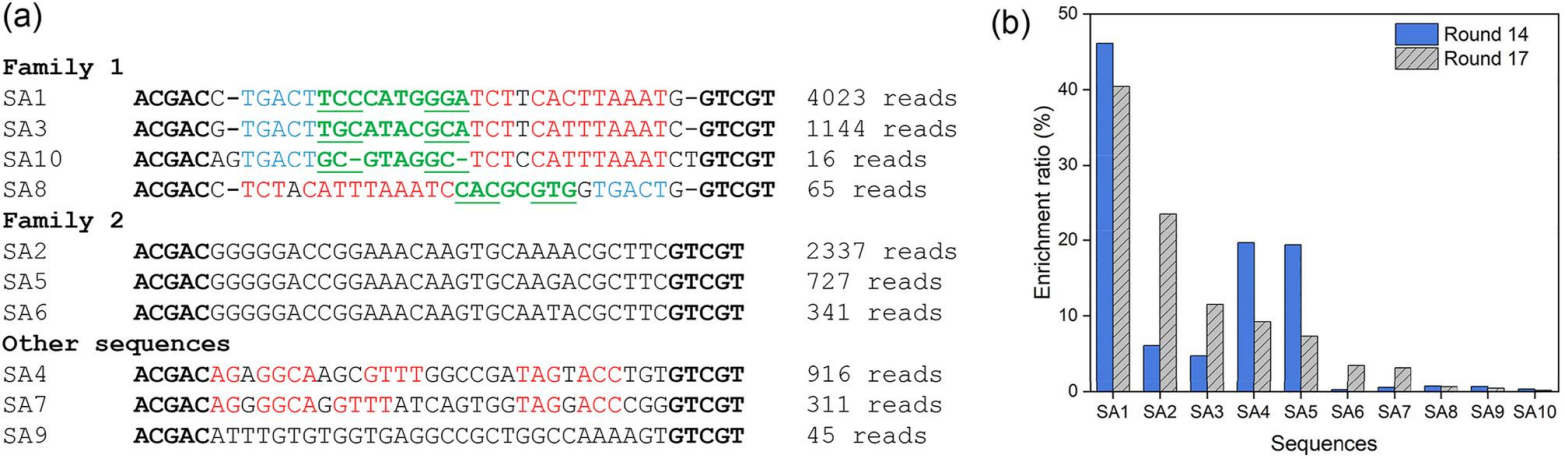
(a) Alignment of the top 10 most abundant sequences from the round 17 selection. Two major families were identified. The nucleotides from the primer‐binding regions are in black boldface. Conserved regions are shown in blue and red. Some hairpin motifs are marked in green and the underlined nucleotides can form stems. (b) Abundance of the top 10 round 17 sequences in round 14.

Figure 1a shows the alignment of the top 10 most abundant sequences in round 17. We identified two main families. Family 1 (represented by SA1) is the dominating family containing two highly conserved regions highlighted in blue and red, respectively, which are separated by a small hairpin in green. The hairpin sequence is highly variable, but the stem‐loop structure is retained, indicating that the hairpin is not directly involved in target binding.^[26]^ We also observed that SA8 in family 1 has circular permutation sequences for the two conserved regions, although SA8 has very low abundance. Such a sequence feature is commonly found in small molecule binding aptamers.[^22^, ^27^, ^28^] Family 2 (represented by SA2) has a very long conserved region, with the three sequences in this family differing only by one nucleotide. The family 2 sequences are rich in purine. The remaining three sequences did not appear to belong to either family. Among them, two of the sequences have a lot of common regions, but they are of relatively low abundance. To make sure that we did not miss important aptamers, we also picked SA4 for further analysis.

We then compared the sequence distribution in rounds 14 and 17 (Figure 1b). The topmost sequence remained the same, although the relative abundance of the next few sequences varied. Nevertheless, the top five sequences remained the same in these two rounds, indicating that the library was already highly converged even at round 14 before we dropped the concentration of SA. The fact that the most abundant sequence remained indicated that this sequence might be the best candidate for binding to both high and low concentrations of SA. The high sequence convergence indicated a successful aptamer selection.

### 3.2 ThT‐based binding assays

**FIGURE 2:**
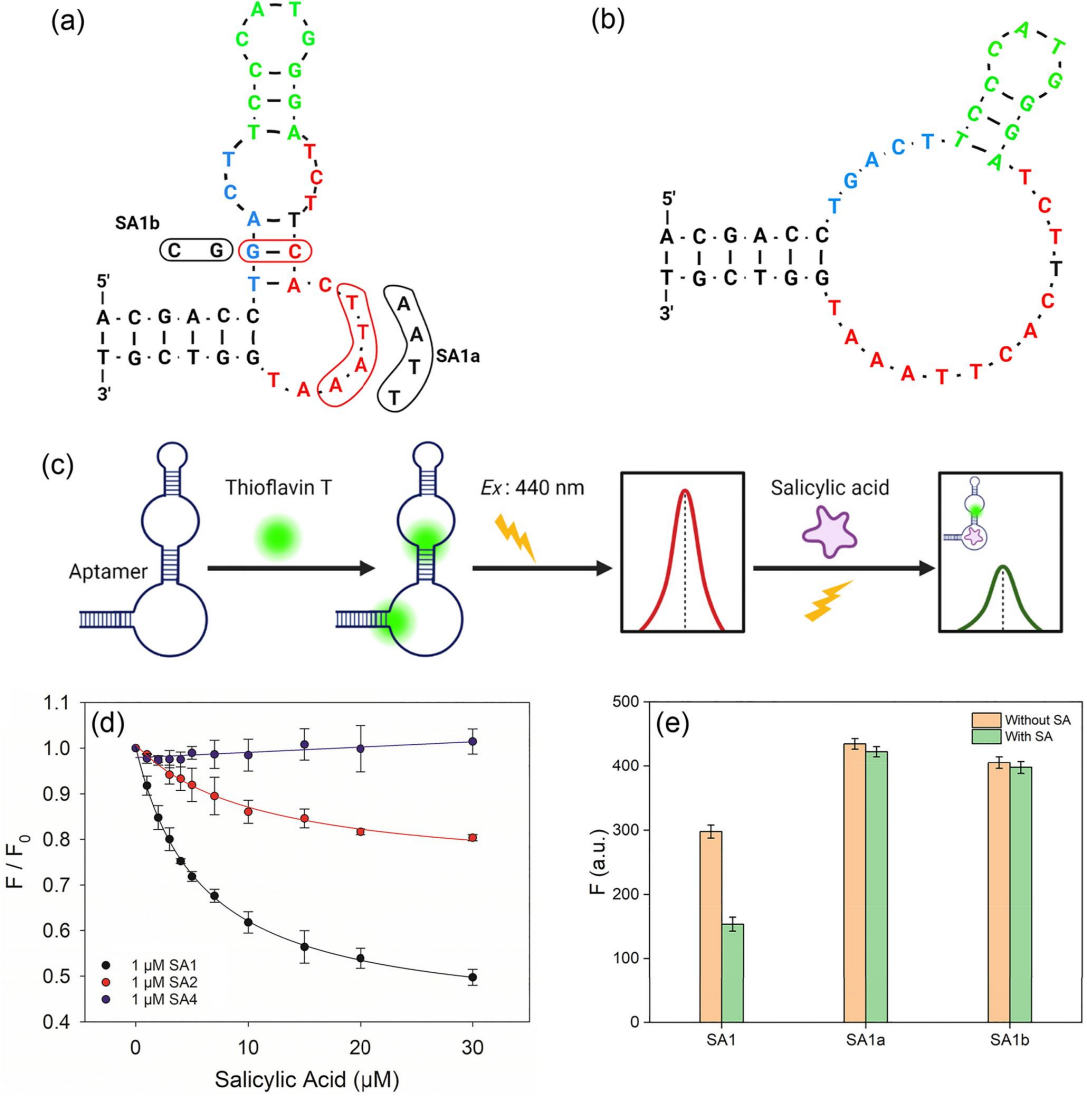
The secondary structure of SA1 (a) predicted by Mfold and (b) based on mutation studies. (c) Scheme of thioflavin T (ThT) fluorescence assay. (d) Titration curve of three aptamers from the three families. (e) Fluorescence change of the mutants of SA1. The assays were performed using 1 μM DNA with 2 μM ThT in selection buffer, pH 6.0 with 100 mM NaCl and 1 mM MgCl_2_.

To evaluate binding of the aptamers, we chose SA1,SA2 and SA4 for further studies since they belonged to different families and had the highest abundance in their respective families. SA1 can fold into the structure shown in Figure 2a as predicted by Mfold.^[29]^ We first used ThT as a label‐free dye to probe its binding to SA (Figure 2c).[^30^, ^31^] Even though this SA1 aptamer is not rich in guanine, ThT can still nonspecifically bind to it and enhance the fluorescence. Upon target binding, a fraction of the ThT is displaced and decreased fluorescence is observed. Thioflavin T has been used in many non‐G‐quadruplex aptamers.[^32^, ^33^] We mixed 1 μM SA1 aptamer and 2 μM ThT and then gradually titrated SA. A gradual drop in the ThT fluorescence at 490 nm was observed, and eventually more than 50% fluorescence drop was achieved (Figure 2d, black trace). We fitted the fluorescence change to a one‐site binding model and obtained an apparent dissociation constant (*K*_*d*_) of 5.8 μM. Using the same method, we also tested the SA2 aptamer, for which the fluorescence change was much smaller reaching only around 20%. By fitting the data, we obtained a *K*_*d*_ of 11 μM for SA2. Finally, the ThT fluorescence barely changed for SA4. From a biosensor design standpoint, if we use ThT as a probe, SA1 is the best candidate. Therefore, this study is focused on SA1.

### 3.3 Aptamer secondary structure analysis

The predicted secondary structure of SA1 is shown in Figure 2a. Its two ends are closed by a 6 base pair (bp) stem and the conserved nucleotides are colored in red and blue that are connected by a hairpin in green. Since a 3 bp stem region is predicted in the conserved region, there is a possibility that these base pairs are not real. Otherwise, one would expect the covariation of the bases as long as base pairs are maintained. To understand whether this is a real base paired region or not, we then designed a mutant named SA1a by replacing the middle G‐C pair with a C‐G pair. If this is a paired region, this swapping would not affect SA binding. Using the ThT assay, we found that the mutant showed no binding to SA (Figure 2e). Therefore, this is not a paired region and the actual secondary structure should be as shown in Figure 2b.

To further confirm that the ThT assay can provide specific binding information and specific SA binding, we then designed a mutant by changing the TTAA in one of the conserved regions to AATT. This mutant named SA1b had the same base composition and the same predicted secondary structure. The ThT assay also showed no binding (Figure 2e). Overall, the ThT assay is reliable for understanding the binding and secondary structure of the SA1 aptamer.

In 2019, Chen et al reported a set of SA binding aptamers.^[14]^ Those aptamers were very long (∼91‐nt), which more than doubled the length of our aptamers. We tried to align our SA1, SA2 and SA4 sequences with the previously published aptamers (Figure S1), but the conserved regions were quite different. Therefore, we believe that our aptamers were different from the previously reported ones and were new aptamers.

### 3.4 Salicylic acid binding measured by isothermal titration calorimetry

**FIGURE 3:**
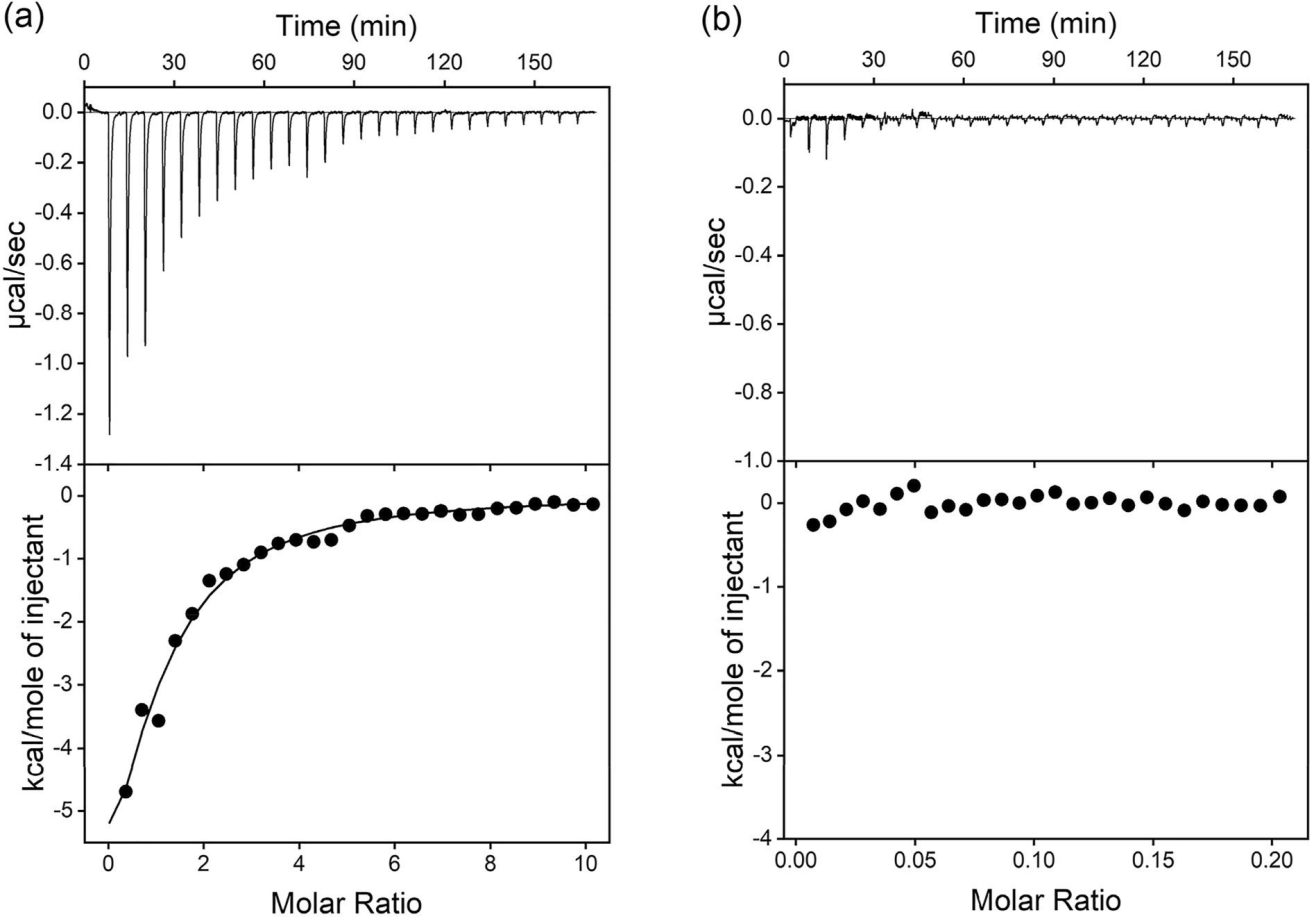
(a) Titration of 1 mM Salicylic acid (SA) into 20 μM SA1 aptamer after background subtraction in MES buffer, pH 6.0 with 5 mM MgCl_2_ and 100 mM NaCl. (b) Isothermal titration calorimetry (ITC) of the background signal by titrating SA into the buffer. MES, 2‐(4‐Morpholino)ethanesulfonic acid.

To further confirm binding, we then used ITC. We titrated 1 mM SA into 20 μM SA1 aptamer and observed an exothermic reaction (Figure 3a). The fitted *K*_*d*_ value from ITC was 26.7 ± 5.6 μM. The background of titrating SA into buffer was negligible (Figure 3b). The binding stoichiometry *N* was fitted to be 0.82, suggesting that each SA1 aptamer binds one SA molecule. The Δ*H* value was −13.7 kcal/mol, and the Δ*S* value was −24.9 cal/K⋅mol. Therefore, this is an enthalpy‐driven binding.

The *K*_*d*_ value from the ITC measurement was four‐fold that from the ThT assay. Isothermal titration calorimetry is a more reliable measurement of aptamer binding since no other components were present to interfere with binding. It is quite common that different binding assays result in different *K*_*d*_ values.[^34^, ^35^] In this particular case, it might be that positively charged ThT favored binding of negatively charged SA to the negatively charged aptamer, resulting in a smaller *K*_*d*_ for the ThT method. Nevertheless, this is a reasonable binding affinity range for a simple target‐like SA. For example, the classical adenosine aptamer has a *K*_*d*_ of 6 μM,^[36]^ and a recently reported chloramphenicol aptamer has a *K*_*d*_ of 9.8 μM.^[37]^ Both are more complex molecules compared to SA.

### 3.5 Effect of metal ions on Salicylic acid binding

**FIGURE 4:**
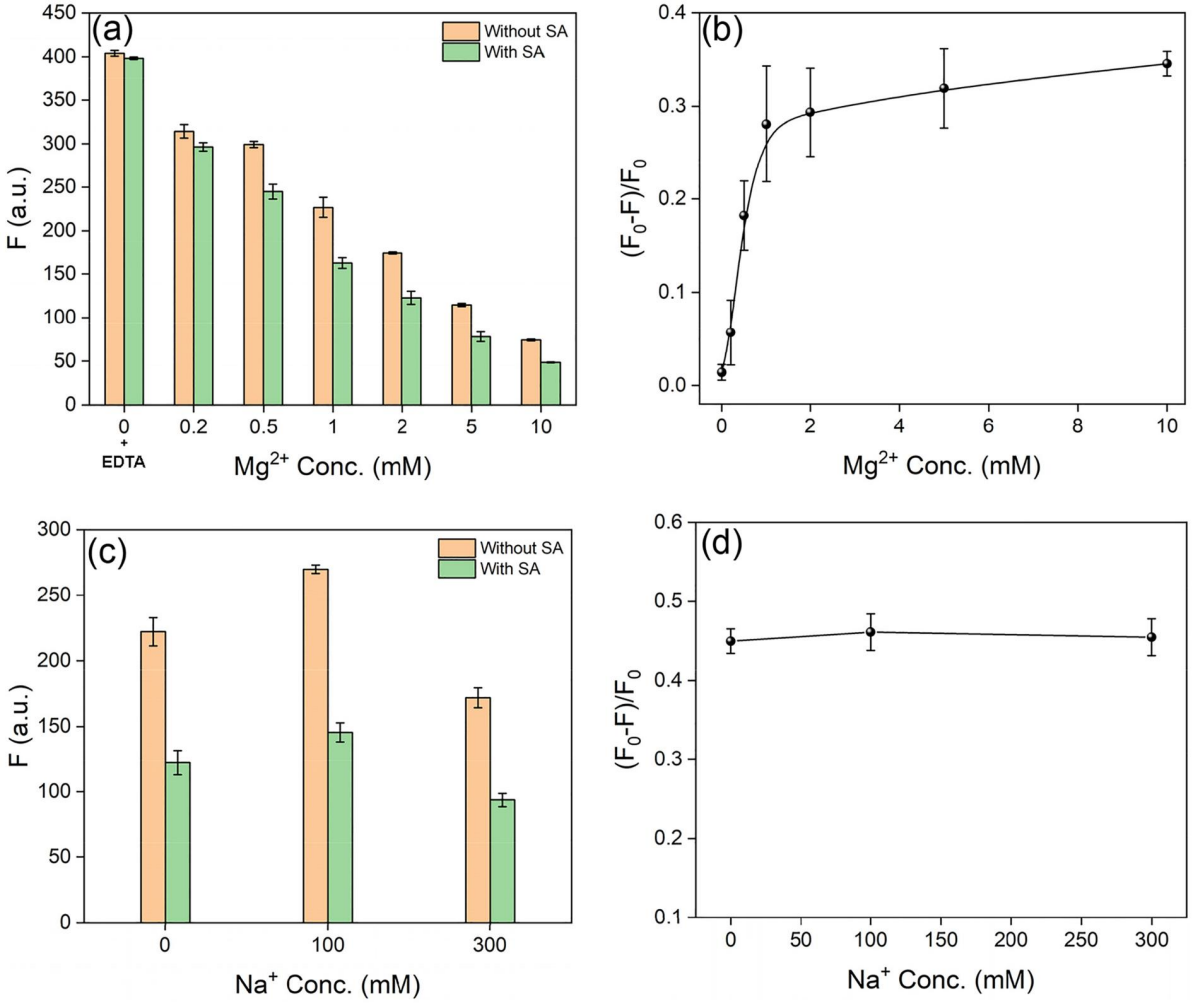
Thioflavin T (ThT) assays for (a, b) Mg^2+^ ‐dependent SA1 aptamer binding in the presence of 100 mM NaCl, and (c, d) Na^+^ dependent binding in the presence of 1 mM MgCl_2_. Both the (a, c) fluorescence values and (b, d) fraction of fluorescence drop are presented.

Aptamer binding is often strongly dependent on metal ions.[^38^, ^39^] Thus, we used the ThT assay to evaluate the effect of salt concentration. First, we fixed the NaCl concentration to be 100 mM and varied the Mg^2+^ concentration up to 10 mM (Figure 4a). Without Mg^2+^, the fluorescence drop disappeared, indicating that the binding required divalent Mg^2+^. To ensure that no divalent metal was present, we also added 5 mM EDTA to this sample. The signal change then drastically increased with increasing Mg^2+^ concentration, and saturation was achieved at 1 mM Mg^2+^ (Figure 4b). In terms of the overall signal intensity, the sample with 10 mM Mg^2+^ dropped significantly, likely due to the screening of electrostatic interactions between ThT and SA1 aptamer by Mg^2+^. Therefore, 1 mM Mg^2+^ was optimal. We then fixed Mg^2+^ concentration at 1 mM and varied the NaCl concentration (Figure 4c,d). For all the samples, the signal changes were similar and the fluorescence intensity at 100 mM NaCl was slightly higher. Therefore, 1 mM Mg^2+^ and 100 mM Na^+^ were determined to be the optimal conditions for the ThT‐based assay.

### 3.6 Highly sensitive and selective sensing of Salicylic acid

**FIGURE 5:**
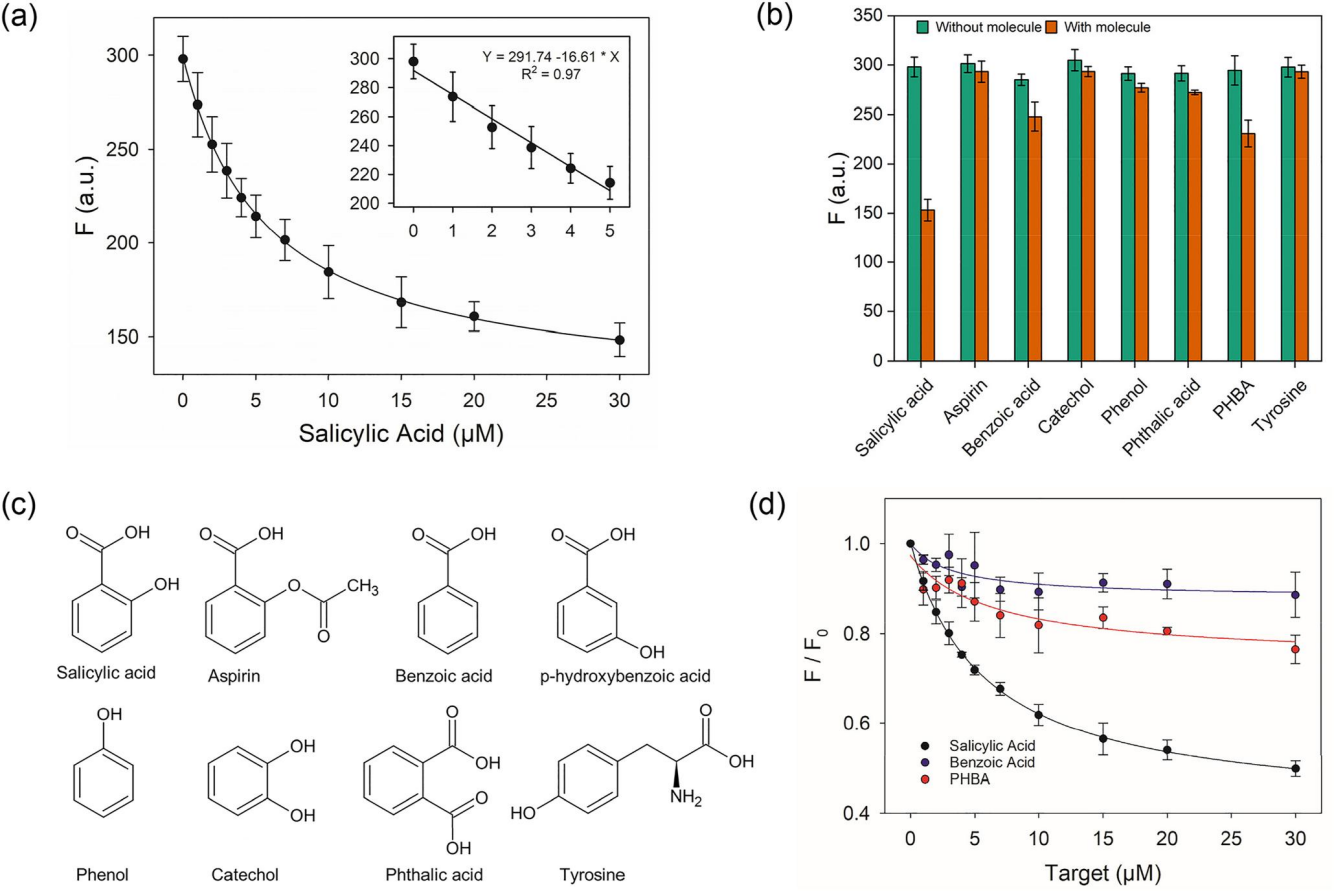
(a) Sensitivity test of the SA1 and ThT‐based Salicylic acid (SA) sensor. Inset: response of the sensor at low SA concentrations. (b) Selectivity test using 30 μM of each molecule. PHBA means p‐hydroxybenzoic acid. (c) Structures of SA and other compounds tested for selectivity. (d) Titration of a few molecules to the sensor.

The above assay used 1 μM SA1 aptamer. We then optimized the concentration of the SA1 aptamer and compared the relative fluorescence change (Figure S2). In principle, a lower SA1 concentration can provide higher sensitivity for detection, but it may suffer from more signal variation at the same time. A higher SA1 concentration gave a stronger initial fluorescence (Figure S2). With 0.1 μM SA1 aptamer, the signal barely changed. Therefore, for the subsequent sensor experiments, we chose to use 1 μM SA1 aptamer. Under the optimized conditions, we then measured the sensitivity of the sensor (Figure 5a). The detection limit was calculated to be 2.2 μM SA based on 3σ/slope, where *σ* is the standard deviation of the background fluorescence variation (inset of Figure 5a).

We then tested the selectivity (Figure 5b). The structures of the related molecules are shown in Figure 5c. Among them, SA showed the highest fluorescence drop. Interestingly, we also observed a slight signal drop with benzonic acid and p‐hydroxy benzonic acid. Importantly, no change was observed with aspirin, indicating that capping the ‐OH group by an acetyl group fully disrupted aptamer binding. For the two molecules that showed a slight signal change, we also performed a titration experiment (Figure 5d), and the better selectivity for SA is obviously observed from these data.

### 3.7 Detection of Salicylic acid in real samples

**FIGURE 6:**
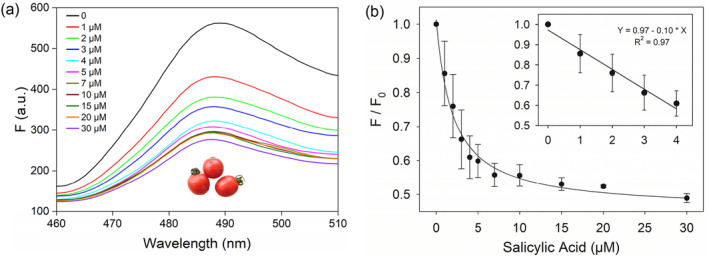
(a) Fluorescence spectra of the sensor in the presence of different Salicylic acid (SA) concentrations dissolved in 10% tomato juice. (b) The calibration curve in 10% tomato juice.

To verify whether our sensor can withstand the stress of a complex environment, we further challenged our sensor in extracted tomato juice. The raw tomato juice contained a large number of complex components which can provide a good reference environment for SA detection in foods. We collected the juice and used 10% raw juice to test the sensor by spiking various concentrations of SA. The results showed that our sensor can recognize SA in this sample matrix (Figure 6a) with a detection limit of 2.8 μM (Figure 6b). Therefore, this sensor has a great application potential in the field of food safety and drug monitoring.

## 4 CONCLUSIONS

In this work, we used the library‐immobilization method to select aptamers for SA. Two families of SA‐binding aptamers were obtained, and the SA1 aptamer in family 1 showed a tighter *K*_*d*_ value. The SA1 sequence had a *K*_*d*_ of 26.7 μM from ITC. Using ThT fluorescence spectroscopy, we measured the *K*_*d*_ of SA1 to be 5.8 μM. Using the ThT assay as a biosensor reaction, we measured the limit of detection to be 2.2 μM SA. Comparisons were made with a set of previously reported SA binding aptamers using sequence alignment. Based on our conserved sequences, the SA1 is a new aptamer. In addition, our aptamers were shorter by more than half and the secondary structure of SA1 was characterized by mutation studies. Salicylic acid is an important analyte in drug and food industry and this aptamer may find useful applications in developing biosensors for SA.

## CONFLICT OF INTEREST STATEMENT

The authors declare no conflicts of interest.

## ETHICS STATEMENT

Not applicable.

## Supporting information

Supplementary Material

## Data Availability

The data that support the findings of this study are openly available in the Federated Research Data Repository at http://doi.org/10.20383/103.0785.
