# Supplementary material for: Capture‐SELEX for a short aptamer for label‐free detection of salicylic acid
Source: Smart Mol. 2023 Aug 28;1(3):e20230007. doi: 10.1002/smo.20230007 (PMC12118186; doi:10.1002/smo.20230007)
Supplement: Supplementary file 1 — Supplementary Material [file SMO2-1-e20230007-s001.pdf]

Table S1. DNA sequences used for aptamer selection and assays.

DNA Names	Sequence and modification (From 5′ to 3′)
N30 Library	GGAGGCTCTCGGGACGAC**N30**GTCGTCCCGACTCTATGATGACTGT
Forward primer	GGAGGCTCTCGGGACGAC
Reverse primer	ACAGTCATCATAGAGTCGGGACG
Biotin-reverse primer	/**5Biosg**/ACAGTCATCATAGAGTCGGGACG
Biotin-column	GTCGTCCCGAGAGCCATA/**3BioTEG**/
SA1	ACGACCTGACTTCCCATGGGATCTTCACTTAAATGGTCGT
SA1a	ACGACCTGACTTCCCATGGGATCTTCACAATTATGGTCGT
SA1b	ACGACCTCACTTCCCATGGGATCTTGACTTAAATGGTCGT
SA2	ACGACGGGGGACCGGAAACAAGTGCAAAACGCTTCGTCGT
SA4	ACGACAGAGGCAAGCGTTTGGCCGATAGTACCTGTGTCGT

Note: /5Biosg/ is biotinylation at the 5′-end, and /3BioTEG/ is biotinylation at the 3′-end with extended spacer

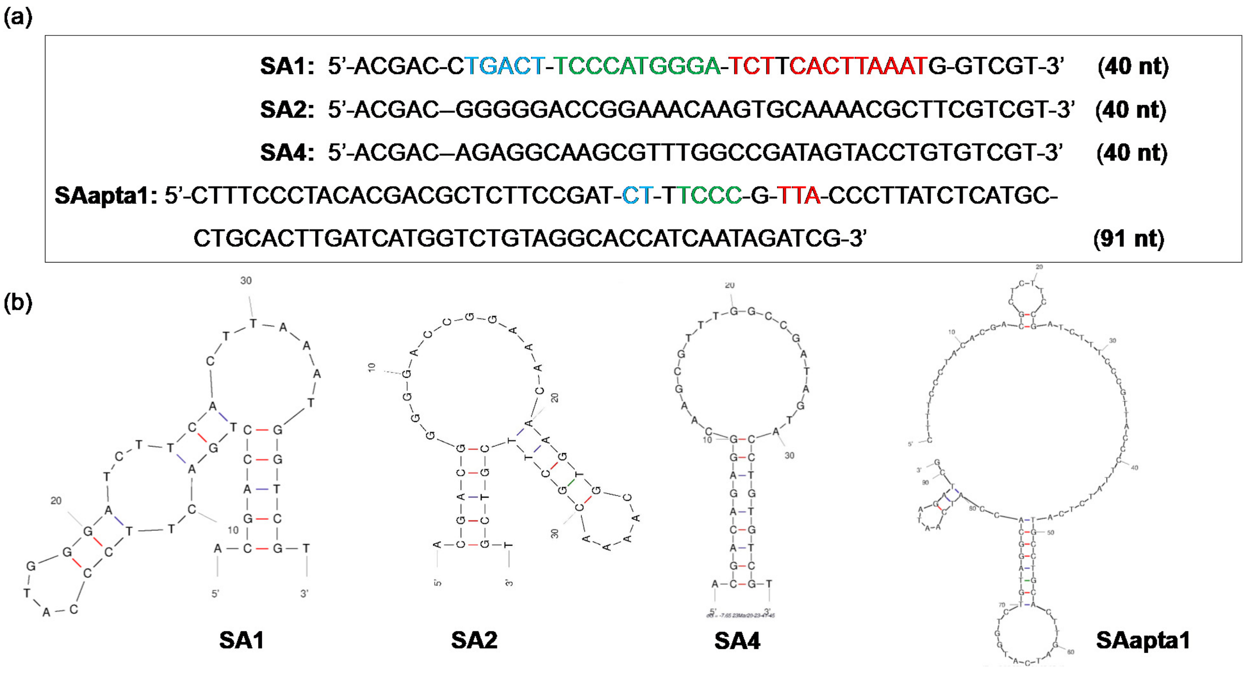

Figure S1. (a) The alignment of the SA1, SA2, SA4 (from this work) and SAapta1 (from a previous publication) aptamers. The conserved regions in SA1 are shown in blue and red, and some of the common nucleotides in SAapta1 are also marked. The hairpin sequences was marked in green. (b) The secondary structure of SA1, SA2, SA4 and SAapta1 sequences predicted by Mfold. Based on the primary sequence and secondary structure, SA1 and SAapta1 are different aptamers.

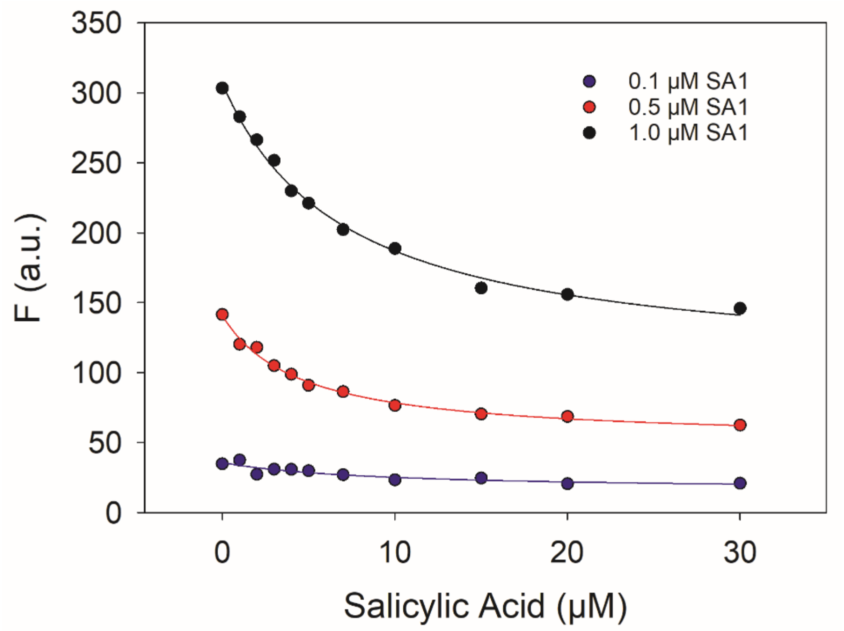

Figure S2. Optimization of the SA1 aptamer concentration. For this experiment, the ThT concentration was fixed at 2 μM in MES buffer, pH 6.0 with 1 mM $MgCl_2$ and 100 mM NaCl.
